# Activating transcription factor 3 is overexpressed in human glioma and its knockdown in glioblastoma cells causes growth inhibition both *in vitro* and *in vivo*

**DOI:** 10.3892/ijmm.2015.2173

**Published:** 2015-04-07

**Authors:** SIQI MA, CHANGHE PANG, LAIJUN SONG, FUYOU GUO, HONGWEI SUN

**Affiliations:** Department of Neurosurgery, The First Affiliated Hospital of Zhengzhou University, Zhengzhou, Henan 450052, P.R. China

**Keywords:** activating transcription factor 3, mammary serine protease inhibitor, matrix metalloproteinase 2, siRNA, glioblastoma

## Abstract

Glioblastomas are highly malignant gliomas that are extremely invasive with high rates of recurrence and mortality. It has been reported that activating transcription factor 3 (ATF3) is expressed in elevated levels in multiple malignant tumors. The purpose of this study was to investigate the function of ATF3 in the development of glioma and its clinical significance. Immunohistochemical staining, western blot analysis and RT-qPCR revealed that the mRNA and protein levels of ATF3 and matrix metalloproteinase 2 (MMP2) were higher in the glioma than in the normal human brain tissues, and that their levels were proportional to the pathological grades. By contrast, the mRNA and protein levels of mammary serine protease inhibitor (maspin; SERPINB5) were significantly lower in the glioma than in the normal brain tissue, and maspin expression was inversely proportional to the glioma pathological grade. The transfection of U373MG glioblastoma cells with ATF3-siRNA induced a number of changes in cell behavior; the cell proliferative activity was decreased and flow cytometry revealed an increased proportion of cells arrested in the G_0_/G_1_ phase of the cell cycle. In addition, TUNEL staining indicated an increased proportion of cells undergoing apoptosis and Transwell assays revealed impaired cell mobility. The sizes of the tumors grown as xenografts in nude mice were also significantly reduced by treatment of host mice with ATF3-siRNA. Taken together, these results suggest that ATF3 promotes the progression of human gliomas.

## Introduction

Gliomas are the most common primary tumors of the central nervous system. The unique biological characteristics of glioma cells, such as the invasiveness to surrounding tissues, the difficulty of complete resection and the high probability of recurrence *in situ* are important topics in present study. The invasion of glioma cells into the surrounding tissue is a complex process involving multiple steps, including the adherence and migration of tumor cells and the degradation of the extracellular matrix. Previous studies have demonstrated that activating transcription factor 3 (ATF3) is highly expressed in several malignant cancer tissues (1–3). ATF3 can induce cells to enter the cell cycle from the stationary phase, thus accelerating cell proliferation; this characteristic is important in the processes of invasion and migration and is significant for the prognosis for several types of tumor (4–6). Maspin (SERPINB5) is a tumor suppressor gene that suppresses angiogenesis, enhances the ability of cells to adhere and suppresses cancer cell migration (7). Another important factor in glioma invasion is matrix metalloproteinase 2 (MMP2), which has been reported to destroy local tissue and enhance tumor angiogenesis, thereby accelerating glioma invasion and migration (8). In the present study, we employed immunohistochemical staining, western blot analysis and RT-qPCR to assess the protein and mRNA expression levels of ATF3, maspin and MMP2 in human brain glioma samples. We then conducted a series of experiments using the human glioblastoma cell line, U373MG, in which the cells were transfected with ATF3-siRNA or a control in order to assess the cell proliferative capacity, cell cycle status and apoptotic fraction, as well as the ability of the cells to invade through fibronectin. We also used immunocytochemistry, RT-qPCR and western blot anlaysis to assess the changes in the protein and mRNA expression of ATF3, maspin and MMP2 in cultures of U373MG cells *in vitro*. Finally, we determined the ability of ATF3-siRNA to inhibit the growth of U373MG cells grown *in vivo* as subcutaneous xenografts in nude mice. The objective was to elucidate the role of ATF3, maspin and MMP2 in the development of gliomas.

## Materials and methods

### Human tissues

Astrocytoma samples that were resected during surgery from September 2008 to December 2009 at the First Affiliated Hospital of the Medical College of Zhengzhou University were collected. All patients provided signed informed consent and the study was approved by the Research Ethics Committee of Zhengzhou University.

Material from 100 glioma cases (58 males) was examined. The age range was 18–66 years, with an average age of 42.3±3.1 years (SD). All pathological sections were analyzed by two experienced pathologists. Cases were graded according to the WHO classification criteria in 2007 (9) for central nervous system tumors: 15 cases were grade I (pilocytic astrocytoma), 32 cases were grade II (diffuse astrocytoma), 30 cases were grade III (anaplastic astrocytoma) and 23 cases were grade IV (glioblastoma multiforme). Thirteen control brain tissue samples (8 males and 5 females) were available from resection during surgery from patients with craniocerebral trauma in the same hospital during the same time period; the control samples were proven pathologically to be normal brain tissues. From each tumor patient, two samples of central, fresh tumor tissue without bleeding or necrosis were stored in liquid nitrogen, and another sample was fixed with 10% formalin, embedded in paraffin and cut into 5-*μ*m-thick sections for hematoxylin and eosin and immunohistochemical staining.

### Main reagents

The rabbit polyclonal anti-human ATF3 (sc-188), anti-maspin (sc-22762) and anti-MMP2 (sc-10736) antibodies, the reference gene β-actin, goat anti-rabbit IgG antibody and the DAB kit, as well as chemiluminescence reagents used for western blot analysis were purchased from Santa Cruz Biotechnology, Inc. (Santa Cruz, CA, USA). All PCR primers were designed and synthesized by Shanghai Sangon Biological Engineering Technology & Services Co., Ltd. (Shanghai, China). The PureLink^®^ RNA Mini kit (12183018A), the high-capacity cDNA reverse transcription kit (4368813), Power SYBR^®^-Green PCR Master Mix (4367659), the pSilencer2.1 U6 vector and Lipofectamine 2000 used for transfection were purchased from Invitrogen (Life Technologies, Carlsbad, CA, USA). The U373MG cells were obtained from the American Type Culture Collection (ATCC^®^ HTB-17; ATCC, Rockefeller, MD, USA) in 2010; however, there is a possibility that this glioblastoma cell line should be described as another glioblastoma cell line, U-251 MG, as similarities have been reported between these two cell lines (http://www.lgcstandards-atcc.org/support/faqs/cf245/U373%20MG%20ATCC%20HTB17-1055.aspx). MTT tetrazolium substrate, propidium iodide (PI) for flow cytometry and terminal deoxynucleotidyl transferase-mediated dUTP nick-end labeling (TUNEL) reaction mixture were from Roche Diagnostics (Laval, Quebec, Canada). The Transwell cell culture inserts were obtained from Corning Inc. (Corning, NY, USA).

### Immunohistochemistry

Sections of paraffin-embedded tissue were deparaffinized in dimethylbenzene and hydrated using an alcohol gradient. The sections were incubated in 0.3% H_2_O_2_ in methanol for 30 min at room temperature and in an ice bath for 5 min in 0.1% Triton X-100, and blocked with 10% goat serum. The working dilutions of primary antibodies against ATF3, maspin and MMP2 in the tissue sections were 1:200. The development of color using DAB-H_2_O_2_ was observed under a CX 31 Olympus microscope (Olympus Optical Co., Ltd., Tokyo, Japan). Counterstaining was performed with hematoxylin, and following dehydration, the sections were coverslipped with neutral gum. Normal goat serum was used as a substitute for the primary antibody in the negative control group and tissue sections known to be positive were used as positive controls. Obvious brownish yellow particles observed in the nucleus or cytoplasm indicated positive staining for ATF3, maspin and MMP2 proteins. We used the semi-quantitative judgment method of Gatalica *et al* (10) for the analysis of the experimental results by the H-score (H=I × P) system. Five high-power fields (×400, final magnification) were selected for each section. The average positive rates were calculated and were expressed as the means ± SD.

### Measurement of mRNA levels by RT-qPCR

Total RNA extraction, the reverse transcription of mRNA into cDNA and fluorescence quantitative PCR (qPCR) were performed according to the manufacturer’s instructions. We used β-actin (ACTB) mRNA as an internal reference. The forward primer for ATF3 was 5′-CCTCGGAAGTGAGTGCTTCT-3′ and the reverse primer was 5′-ATGGCAAACCTCAGCTCTTC-3′. The forward primer for maspin was 5′-AGACATTCTCGCTTCCCTGA-3′ and the reverse primer was 5′-AATTTTGACCCCTTATGGGC-3′. For MMP2, the forward primer was 5′-GCTATGGACTTGGGAGAA-3′ and the reverse primer was 5′-TGGAACGGAATGGAAAC-3′. The forward primer for β-actin was 5′-CACCACCATGTACCCTGGCA-3′ and the reverse primer was 5′-GCTGTCACCTTCACCGTTCC-3′. The reaction conditions for qPCR were as follows: 95°C for 5 min and 40 cycles of 95°C for 15 sec, 60°C for 1 min, and 72°C for 1 min. For each sample, 3 replicates were assessed and a parallel reaction without primers was used as a negative control. Relative mRNA expression levels were calculated for each gene following normalization against β-actin, using the ΔΔCt method, as previously described (11).

### Measurement of protein levels by western blot analysis

Tissues removed from storage at −80°C, or cells collected from culture were extracted by grinding in RIPA lysis buffer in a glass homogenizer and incubated on ice for 1 h. The extracts were centrifuged for 10 min at 18,000 rcf and the supernatants were collected and mixed with 2X sodium dodecyl sulfate (SDS) loading buffer. Following denaturation for 5 min at 100°C and centrifugation for 10 min at 16,000 rcf, the lysates were electrophoresed on 10% SDS-polyacrylamide gels. A semi-dry western blot analysis transfer method was used. The membranes were blocked with 5% non-fat milk powder for 1 h at room temperature, washed 3 times (5 min each) with TBS-T, incubated with primary antibodies (the working dilutions were 1:1,000) overnight at 4°C, washed 3 times (5 min each) with TBS-T, incubated with secondary antibodies for 2 h at 37°C and washed 3 times (5 min each) with TBS-T. The presence of immunoreactive protein was detected using an ECL method to expose X-ray film. Processed films were scanned or photographed, and the integrated optical densities of the bands were analyzed using a LabWorks 4.0 gel image analysis system. The ratios of the integrated optical densities of the proteins to β-actin were calculated.

### Cell culture

The frozen human glioblastoma cell line, U373MG, was inoculated onto a 25 cm^2^ culture flask after being revived in a 37°C water bath. Low-glucose Dulbecco’s modified Eagle’s medium (DMEM) with 10% heat-inactivated fetal calf serum was added and the cells were placed in an incubator with 5% CO_2_ and saturating oxygen at 37°C. The medium was replaced every other day until the cells spread onto the bottom of the flask. When the cell fusion state was about to occur, the nutrient solution was discarded and the cells were digested by 0.25% pancreatin. When the cells retracted, the intercellular space expanded and the cells became round, the nutrient solution was added to terminate the digestion and the cells were transferred and then centrifuged at 1,100 rcf for 5 min. The supernatant was discarded and the low-sugar DMEM nutrient solution was added. The cells were transferred to a new culture bottle and the bottle was then placed in an incubator with a saturation humidity of 5% CO_2_ at 37°C for cultivation. After 2–3 days, the cells were passaged when the cell convergence degree was at 80–90%. The third generation cells were used for the experiments.

### Screening and identification of ATF3-siRNA

Control and ATF3 hairpin loop siRNAs were cloned using the expression vector, pSilencer2.1 U6. Gene-specific siRNA hairpin loops targeting a 19-nucleotide sequence within human ATF3 were designed and synthesized by Shanghai Sangon Biological Engineering Technology & Services Co., Ltd. Initially, 3 ATF3-siRNAs were tested to identify optimal ATF3-siRNA(s). As a result, 2 of 3 siRNA sequences (5′-GAGCTGAGGTTTGCCATCC-3′ and 5′-GAGGCGACGAGAAAGAAAT-3′) were shown to be useful for ATF3 knockdown. An additional siRNA vector (5′-GCACCACGTGACGGAGCGT-3′) was used as a negative control. The ATF3 and control siRNA sequences contained the *Bam*HI and *Hin*dIII restriction sites included in the forward and reverse strands, respectively. The synthesized oligonucleotides were annealed and inserted into pSilencer2.1 U6 using the *Bam*HI and *Hin*dIII sites. The hairpin siRNA sequences were confirmed by nucleotide sequence analysis. The U373MG cells were then transfected with pSilencer2.1 U6-ATF3 or control siRNAs using Lipofectamine 2000 according to the manufacturer’s instructions. Three days following transfection, cell lysates were harvested and processed for western blot analysis to detect the protein expression of ATF3.

### Cell groups

In the present study, the cells were divided into 5 experimental groups as follows: i) the control group: untreated U373MG cells; ii) the control-siRNA group: the U373MG cells were transfected with control siRNAs; iii) the ATF3-siRNA group: the U373MG cells were transfected with pSilencer2.1 U6-ATF3; iv) the cisplatin group: the U373MG cells were treated with cisplatin; and v) the ATF3-siRNA + cisplatin group: the U373MG cells transfected with pSilencer2.1 U6-ATF3 were treated with cisplatin.

In addition, a positive and negative control group were used. In the positive control group, the cells were treated as follows: the cells were fixed with 4% paraformaldehyde at room temperature for 1 h followed by 2 washes (5 min each) with PBS. The cells were then treated with 0.1% Triton X-100 for 5 min at room temperature followed by 2 washes (5 min each) with PBS. This was followed by the addition of 100 *μ*l DNase I reagent (2,000 U DNase I, 40 mM Tris-HCl pH 7.9, 10 mM NaCl, 6 mM MgCl_2_ and 10 mM CaCl_2_) and treatment for 30 min at room temperature (37°C) followed by 3 washes (5 min each) with PBS. TUNEL reagent (50 *μ*l) was then added to each well and the remaining steps were the same as those of the experimental procedure in this study. The cells in the negative control group was treated as follows: the cells were fixed with 4% paraformaldehyde at room temperature for 1 h followed by 2 washes (5 min each) with PBS. The cells were then treated with 0.1% Triton X-100 for 5 min at room temperature followed by 2 washes (5 min each) with PBS. This was followed by the addition of 50 *μ*l labeling buffer (5X 550 *μ*l, with reagent containing nucleotide mixture) without DNase I buffer to replace the TUNEL reagent and the cells were incubated at 37°C in a dark, humid environment for 60 min before being washed 3 times with PBS. DAPI solution buffer was added and the following steps were the same as those of the experimental procedure in this study. The addition of DNase I in the positive control group made the cell sample produce DNA double-strand breaks, thus leading to cell apoptosis to the maximum degree, so that the final results presented the maximum positive degree, so as to be used as the positive control. No TUNEL reagent was added to the cells in the negative control group, and thus no apoptotic cells could be tested, and the final result presented complete negative, so as to be used as the negative control. The cells in the control group (untreated group) were not treated at all, so as to test the normal apoptotic rate of the cells.

### Analysis of cell proliferation

Cell numbers were estimated using an assay based on the reduction of the formazan dye MTT by metabolically active cells. Twenty-four hours post-transfection with the plasmids, the cells were harvested, resuspended and seeded in 96-well cell culture plate at a density of 5,000 cells/well. For each time point, each group was set up in triplicate. An MTT assay was undertaken at 24 h (day 1) and then cisplatin (sc-200896; Santa Cruz Biotechnology, Inc.) at a concentration of 20 mg/l was added to the appropriate wells (cisplatin group and the ATF3-siRNA + cisplatin group) followed by further culture for an additional 24 h (day 2), 48 h (day 3), 72 h (day 4) or 96 h (day 5). Cell number was assessed by the addition of 20 *μ*l (5 mg/ml) MTT solution to each well of a plate followed by incubation for 4 h at 37°C with 5% CO_2_. The plates were then centrifuged at 2,000 rpm for 5 min and the supernatants were discarded. The formazan reaction product was extracted into 100 *μ*l DMSO and the absorbance at 490 nm was measured using a spectrophotometer.

### Analysis of cell cycle phase by flow cytometry

Forty eight hours following transfection, the cells were seeded into 6-well plates, cultured for a further 48 h and then rinsed with phosphate-buffered saline (PBS), digested with trypsin-ethylenediaminetetraacetic acid (EDTA), harvested, centrifuged at 450 rcf for 5 min and resuspended twice before fixation by adding dropwise into to 95% ethanol precooled to −20°C for storage. Prior to analysis, the cells were warmed, centrifuged at 450 rcf for 5 min and resuspended twice, then stained with PI (containing RNase A at 50 *μ*g/ml) at room temperature in the dark for 30 min. The DNA content was analyzed by flow cytometry using the CellQuest program (Becton-Dickinson and Co., Franklin Lakes, NJ, USA).

### Analysis of cell apoptotic fraction by TUNEL staining

Forty-eight hours following transfection, the cells in each group were digested, counted and seeded in 96-well plates at a density of 6×10^4^ cells/ml followed by incubation at 37°C with 5% CO_2_ for 24 h. Cisplatin was added to the appropriate wells at 20 mg/l followed by incubation at 37°C with 5% CO_2_ for a further 24 h. The cells were then fixed with 4% paraformaldehyde at room temperature for 1 h followed by 2 washes (5 min each) with PBS. The cells were treated with 0.1% Triton X-100 for 5 min at room temperature followed by 2 washes (5 min each) with PBS. TUNEL reagent (50 *μ*l) was added to each well and the cells were incubated at 37°C in a dark, humid environment for 60 min before washing 3 times with PBS. DNA was stained by the addition of 50 *μ*l 4′,6-diamidino-2-phenylindole (DAPI) solution (0.01 mg/ml) to each well, incubating at room temperature in the dark for 5 min followed by washing 5 times with PBS. The cells were observed under a fluorescence microscope (IX73-F22FL/PH; Olympus Optical Co., Ltd) and images were captured using the OpenLab 4 imaging program (Perkin Elmer Co., Ltd., Waltham, MA, USA).

### Analysis of cell invasion

The ability of the glioblastoma cells to migrate through fibronectin was assessed using a Transwell culture system. Transfection was performed according to the protocol described above. Cisplatin at 20 mg/l was added to the appropriate wells at 48 h after transfection. The cells were placed in an incubator at 37°C with 5% CO_2_ for 24 h. Subsequently, 10 *μ*l of fibronectin and 50 *μ*l of Matrigel were coated on the Transwell membranes. The cells in each group were digested and counted. In total, 10^5^ cells were placed into a 1.5 ml microcentrifuge tube and centrifuged for 5 min at 450 rcf; the supernatants were removed and 200 *μ*l of medium without serum were added, and the cells were resuspended before being added to the wells in the Transwell plate. Endothelial cell medium was added to the lower wells in the plate and cultured for 24 h in an incubator at 37°C. The Transwell insert was removed from the chamber, the cells remaining in the insert chamber were removed by gentle wiping with a swab and the residues gently washed off with PBS. A mixture of methanol and glacial acetic acid (3:1 v/v; 30 min) was used to fix cells that had moved through onto the lower side of the Transwell insert and the cells were stained by immersing the inserts in 0.1% Crystal violet dye (sc-207460; Santa Cruz Biotechnology, Inc.) for 15 min. The total number of cells that penetrated the Transwell was counted.

### Growth of glioblastoma cells as tumor xenografts in nude mice

These experiments were approved by the Institutional Animal Care and Use Committee at the First Affiliated Hospital of Zhengzhou University (Zhengzhou, China). BALB/c nude mice (n=40), of the female gender, 4–5 weeks old, weighing 15–20 g, were purchased from Hunan SJA Laboratory Animal Co., Ltd. (Changsha, China). The nude mice were raised in isolation cages with independent ventilation at 24–26°C and with free access to water and food. All cages, bedding materials, drinking water and feed were sterilized and surgical procedures were carried out aseptically by experienced personnel.

The U373MG cells were cultured using standard methods. The cells were digested with 0.2% trypsin for 3 min until they reached tge log growth phase and digestion was terminated by the addition of complete culture medium of low-glucose DMEM. The cells were triturated to yield single cell suspensions and washed twice with complete DMEM medium prior to resuspension in PBS. The cells were adjusted to a density of 1×10^7^ cells/ml and 0.2 ml was injected subcutaneously in the back of each nude mouse. All procedures were undertaken in a laminar flow hood to minimize infection.

The growth of the tumors was monitored regularly and the size of each tumor was measured using a vernier caliper every 5 days. The longest (a) and shortest diameter (b) were measured and the approximate volume of the tumor was calculated using the following formula: transplanted tumor size (V) = longest diameter (a) x square of the shortest diameter (b^2^)/2.

When the tumors attained an average volume of 100 mm^3^, the 40 nude mice were weighed and randomly allocated into 5 groups receiving the following injections: i) the vehicle control group: 0.6 ml saline; ii) the control-siRNA group: 0.5 ml irrelevant siRNA (5′-GAAGAAGGAGAAGACGGAG-3′) plus 0.1 ml saline; iii) the ATF3-siRNA group: 0.5 ml ATF3-siRNA fragment (300 nM) plus 0.1 ml saline; iv) the cisplatin group: 0.1 ml cisplatin solution (500 ng/ml) plus 0.5 ml saline; and v) the ATF3-siRNA + cisplatin group: 0.5 ml ATF3-siRNA fragment and 0.1 ml cisplatin solution. These treatments were administered 10 times by peritumoral injection every other day. The tumor inhibition ratio (%) = (average tumor weight in the control group - average tumor weight in the treatment group)/average tumor weight in the control group x100 was calculated for each treatment.

### Statistical analyses

The data were processed with Statistical Product and Service Solutions 18.0 software (IBM, Armonk, NY, USA). Two samples of rank data were compared using the Mann-Whitney U test. Multiple sample comparison of the rank data was carried out using the Kruskal-Wallis H test. The expression of ATF3, maspin and MMP2 in the patient glioma samples of variable grades was analyzed by Spearman’s rank correlation analysis. The correlation of the expression of ATF3, maspin and MMP2 with the pathological grade was analyzed by Spearman’s rank correlation analysis. The mean values of multiple samples were compared by one-way ANOVA. The significance level was set at P<0.05.

## Results

### Immunohistochemical analysis of ATF3, maspin and MMP2 in normal brain tissues and glioma tissues of each histological grade

**Figure 1:**
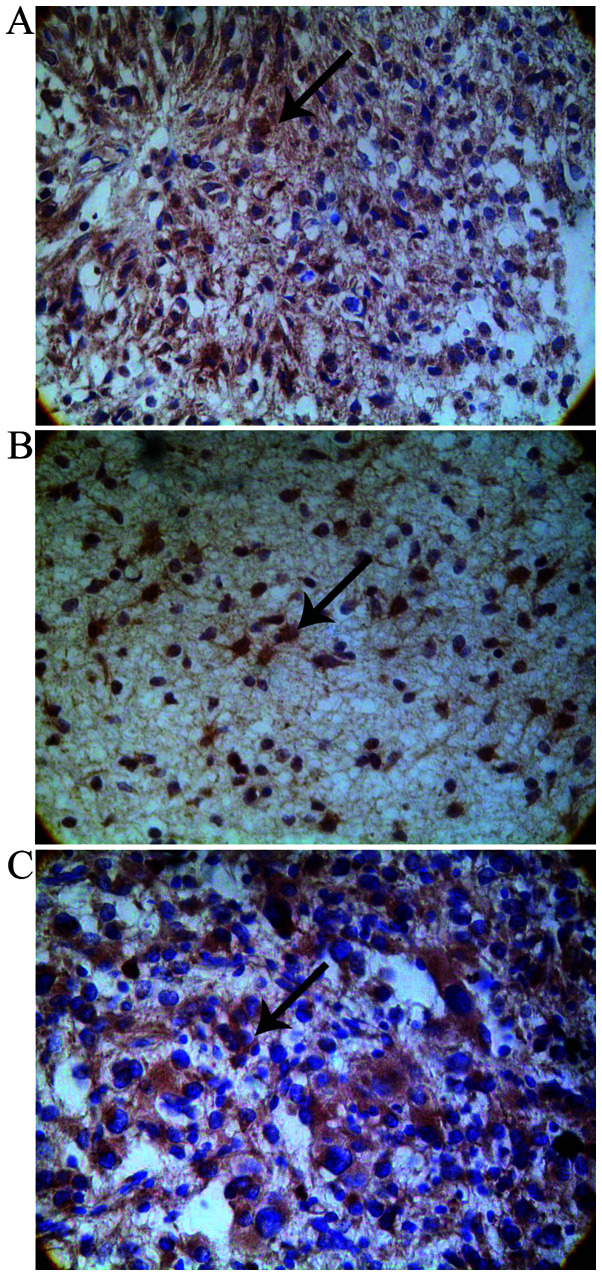
Distribution and expression of activating transcription factor 3 (ATF3), maspin and matrix metalloproteinase 2 (MMP2) in human glioma tissue. Brown staining and particles reflect protein expression (immunohistochemical staining, ×400 magnification). (A) In WHO-defined grade IV gliomas, strongly positive ATF3 staining was mainly located in the tumor cytosol in a diffuse distribution pattern with minor staining of the nuclei (as indicated by arrowhead). (B) In WHO-defined grade I gliomas, maspin was strongly expressed and was mainly located in the cytoplasm and/or nucleus (as indicated by arrowhead). (C) In WHO-defined grade IV gliomas, MMP2 was strongly expressed and distributed mainly in the cytoplasm (less in the nucleus) in a diffuse manner (as indicated by arrowhead).

The presence of ATF3 and MMP2 proteins was mainly manifested as brown particles in the tumor cytoplasm and marginally in the nuclei. They were both irregularly distributed in the lower-grade glioma tissue and diffusely distributed in the higher-grade glioma tissue (Fig. 1A and C). The protein expression of ATF3 and MMP2 in the glioma tissues was evidently higher than that in the normal brain tissues (P>0.05). Their expression in the glioma tissues increased with the increasing glioma grade (I to IV) (Table I). Spearman’s rank correlation analysis revealed that the protein expression of ATF3 and MMP2 positively correlated with the pathological grade of the glioma (ϱ=0.735, P<0.01; ϱ =0.446, P<0.01, respectively).

Maspin protein expression was present both in the nucleus and cytoplasm and it was highly expressed in the normal brain tissues (Fig. 1B). The protein expression of maspin decreased with the increasing glioma grade (I to IV) (Table I) and Spearman’s rank correlation analysis revealed that the protein expression of maspin negatively correlated with the pathological grade of the glioma (ϱ=−0.542, P<0.01).

### Relative abundance of ATF3, maspin and MMP2 mRNA expression in normal brain tissues and glioma tissues of each histological grade

**Figure 2:**
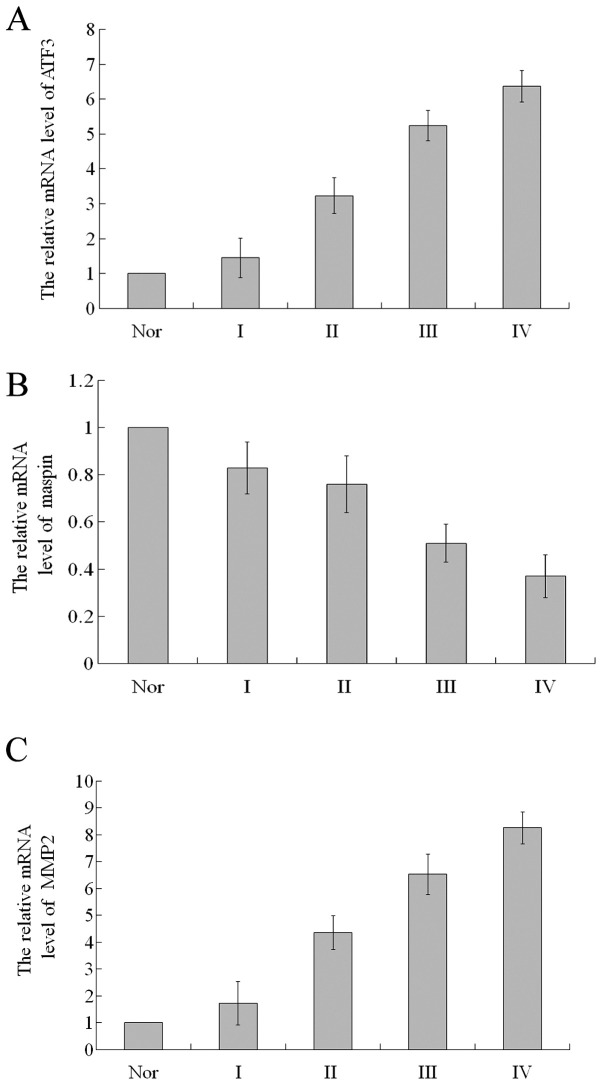
RT-qPCR analysis of the relative mRNA levels for activating transcription factor 3 (AFT3), maspin and matrix metalloproteinase 2 (MMP2). (A) In grade II–IV glioma tissues, ATF3 mRNA expression gradually increased in relative abundance and was higher than that in normal brain tissue (P<0.05). (B) In grade I–IV glioma tissues, maspin mRNA expression gradually decreased and was less abundant compared to normal brain tissue (P<0.05). (C) In grade II–IV glioma tissues, the relative abundance of MMP2 mRNA expression gradually increased and was higher than that in normal brain tissue (P<0.05). Nor, normal tissue.

The relative mRNA expression of ATF3 in the glioma tissues of grade II, III and IV was 3.23±0.51, 5.24±0.43 and 6.37±0.45, respectively, significantly higher compared to the normal brain tissues (all P<0.05; Fig. 2A). The relative mRNA expression of ATF3 in the glioma tissues of grade I did not differ significantly from that in the normal brain tissues (P>0.05). The relative mRNA expression of maspin in the glioma tissues of grade I, II, III and IV was 0.83±0.11, 0.76±0.12, 0.51±0.08 and 0.37±0.09, respectively, significantly lower compared to the normal brain tissues (all P<0.05; Fig. 2B). Similar to the mRNA expression of ATF3, in the glioma tissues of grade I, the relative mRNA expression of MMP2 did not differ significantly from that in the normal brain tissues (P>0.05; Fig. 2C). In the glioma tissues of grade II, III and IV, the relative mRNA expression of MMP2 was 4.36±0.63, 6.53±0.75 and 8.26±0.59, respectively, significanlty higher compared to the normal brain tissues (all P<0.05; Fig. 2C). Spearman’s rank correlation analysis revealed that the mRNA expression of ATF3 and MMP2 positively correlated with the pathological grade of glioma (ϱ= 0.621, P<0.01 and ϱ=0.503, P<0.01, respectively), while the mRNA expression of maspin negatively correlated with the pathological grade of glioma (ϱ=−0.415, P<0.01).

### Western blot analysis of ATF3, maspin and MMP2 protein expression in normal brain tissues and glioma tissues of each histological grade

**Figure 3:**
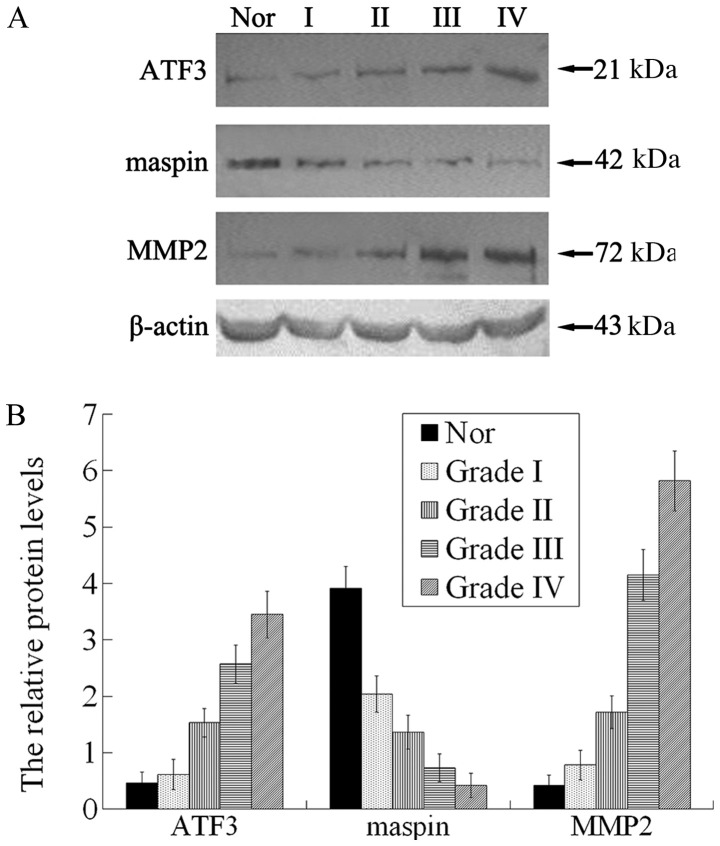
(A) Detection of activating transcription factor 3 (ATF3), maspin and matrix metalloproteinase 2 (MMP2) protein expression in normal brain tissues and all grades of gliomas by western blot analysis. (B) Results of western blot analysis of the relative protein expression of ATF3, maspin and MMP2. In grade II–IV glioma tissues, the protein expression of ATF3 and MMP2 gradually increased and was higher than that in normal brain tissue (P<0.05). In grade I–IV glioma tissues, the protein expression of maspin gradually decreased and was lower than that in the normal brain tissue (P<0.05). Nor, normal tissue.

The intensity of the β-actin bands was similar in all the samples, indicating that the loading of each sample was consistent, and thus that the results are reliable (Fig. 3A). ATF3 protein expression was detected in both the normal brain tissues and glioma tissues of each grade. In the glioma tissue of grade I, the relative protein expression of ATF3 did not differ significantly from that in the normal brain tissues (P>0.05). In the glioma tissue of grade II, III and IV, the relative protein expression was 1.53±0.25, 2.57±0.34 and 3.45±0.41, respectively, significantly higher compared to that in the normal brain tissues (P<0.05; Fig. 3B). In the glioma tissue of grade I to IV, the expression of maspin was 2.04±0.32, 1.36±0.3, 0.73±0.25 and 0.42±0.21, respectively, signficantly lower compared to that in the normal brain tissues (all P<0.05; Fig. 3B). In the glioma tissues of grade I, the protein expression of MMP2 did not differ significantly from that in the normal brain tissues (P>0.05). In the glioma tissues of grade II to IV, the expression levels of MMP2 were 1.72±0.29, 4.15±0.45 and 5.82±0.53, respectively, significantly higher compared to those in the normal brain tissues (P<0.05; Fig. 3B). Spearman’s rank correlation analysis revealed that the protein expression of ATF3 and MMP2 positively correlated with the pathological grade of glioma (ϱ= 0.592, P<0.01 and ϱ=0.726, P<0.01, respectively), while the protein expression of maspin negatively correlated with the pathological grade of glioma (ϱ=−0.517, P<0.01).

### Knockdown of ATF3 using ATF3-siRNA inhibits the proliferative activity of U373MG cells in vitro

**Figure 4:**
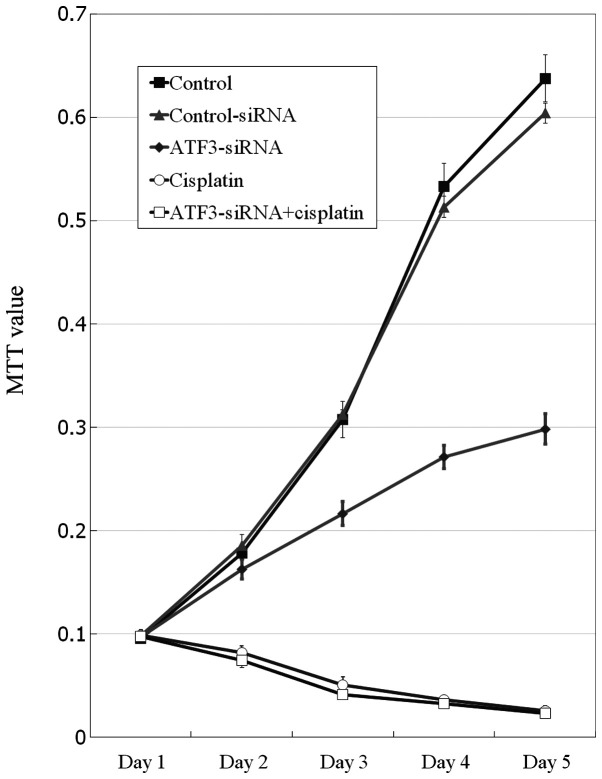
Analysis of cell proliferation by MTT assay. Compared to the cell group and the control-siRNA group, cell proliferation in the activating transcription factor 3 (ATF3)-siRNA group was significantly decreased (P<0.05), and cell proliferation in the cisplatin group and ATF3-siRNA + cisplatin group was reduced more substantially.

Compared with the control group (untreated cells) and the control-siRNA group, the proliferative activity of the cells in the ATF3-siRNA group (transfected with ATF3-siRNA), the cisplatin group (treated with cisplatin) and the ATF3-siRNA + cisplatin group (transfected with ATF3-siRNA and treated with cisplatin) began to decrease by day 2 (P<0.05), and this inhibitory effect occurred in time-dependent manner, i.e. the inhibition intensity increased with time (Fig. 4). Following the addition of cisplatin, the cell proliferative activity gradually decreased with time (P<0.05); no significant difference was observed in the cell proliferative activity between the cisplatin group and the ATF3-siRNA + cisplatin group (P>0.05), which indicated that inhibitory effects of transfection with ATF3-siRNA on the proliferation of U373MG cells were equal to, or as effective to those of treatment with a dose of a chemotherapeutic agent.

### Knockdown of ATF3 using ATF3-siRNA inhibits the cell cycle progression of U373MG cells in vitro

**Figure 5:**
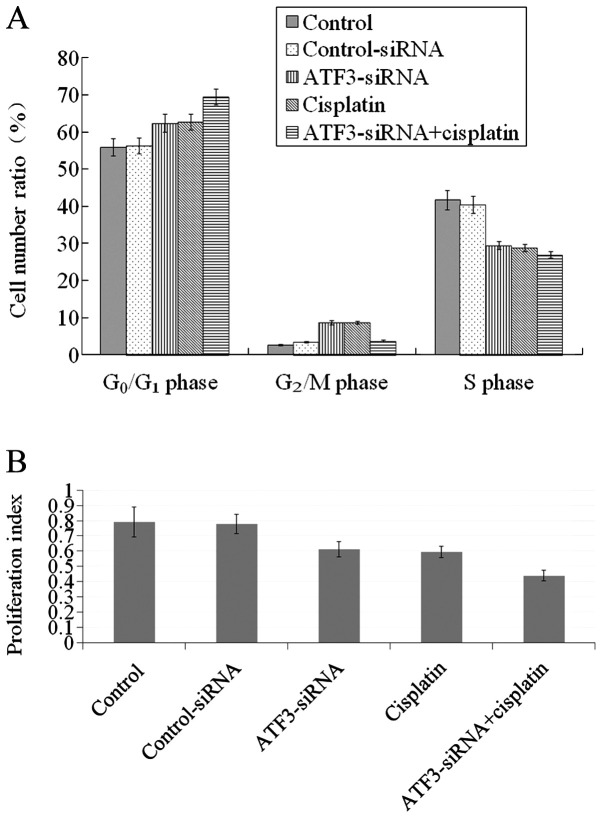
(A) Cell cycle analysis by flow cytometry. Compared to the cell group and the control-siRNA group, the percentage of cells in the S phase in the activating transcription factor 3 (ATF3)-siRNA group and the cisplatin group was lower (P<0.05), whereas the proportion of cells in the G_0_/G_1_ phase was higher (P<0.05). With respect to the ATF3-siRNA group and the cisplatin group, the proportion of cells in the S phase in the ATF3-siRNA + cisplatin group was lower, and the percentage of cells arrested in the G_0_/G_1_ phase was greater (P<0.05). (B) Cell proliferation index. Compared to the cell group and the control-siRNA group, the proliferation index for the ATF3-siRNA group and the cisplatin group was reduced (P<0.05), and the proliferation index in the ATF3-siRNA + cisplatin group was much lower (P<0.05).

The percentage of cells in the S phase in the ATF3-siRNA group and the cisplatin group was 29.42±1.13 and 28.79±0.95%, respectively, which was significantly lower compared to that in the control (untreated) group (41.63±2.55%) and the control-siRNA group (40.36±2.32%) (P<0.05; Fig. 5A). The percentage of cells in the G_0_/G_1_ phase of the cell cycle in the ATF3-siRNA group and the cisplatin group was 62.33±2.35 and 62.63±2.21%, respectively, which was significantly higher compared to that in the untreated control group (55.84±2.23%) and the control-siRNA group (56.27±2.13%; P<0.05). The proliferation indices calculated for the ATF3-siRNA group and the cisplatin group were significantly lower than those for the untreated control group and the control-siRNA group (P<0.05; Fig. 5B). Compared with the ATF3-siRNA group and the cisplatin group, the cells in the ATF3-siRNA + cisplatin group exhibited a lower percentage of cells in the S phase and a higher percentage of cells in the G_0_/G_1_ phase, and had a significantly decreased proliferation index (P<0.05). Following comparisons between the ATF3-siRNA group and the cisplatin group, and between the untreated control group and the control-siRNA group, no significant differences were observed (P>0.05).

### Knockdown of ATF3 using ATF3-siRNA promotes the apoptosis of U373MG cells in vitro

**Figure 6:**
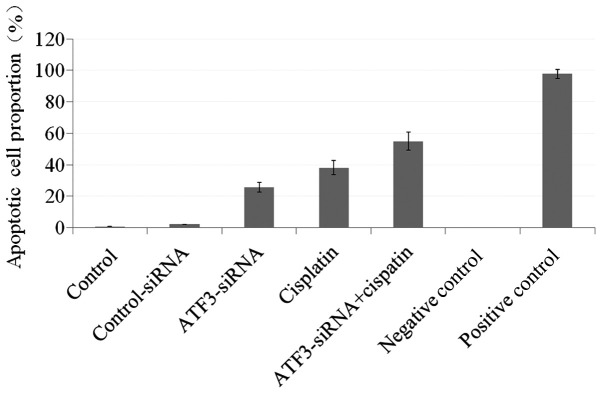
Apoptotic cell index. With respect to the cell group and the control-siRNA group, the proportions of cells in apoptosis in the activating transcription factor 3 (ATF3)-siRNA group, cisplatin group and ATF3 siRNA + cisplatin group were successively increased (P<0.05).

Compared with the untreated control group and the control-siRNA group, the proportion of apoptotic cells in the ATF3-siRNA group, the cisplatin group and the ATF3 siRNA + cisplatin group (25.84±3.107, 38.12±4.543 and 54.89±5.739%, respectively) gradually increased (P<0.05) and intergroup comparisons between these 3 groups revealed significant differences (P<0.05). Apoptotic signals in the negative control group were absent (0%), but were present in virtually all cells in the positive control group (97.72±2.902%; Fig. 6).

### Knockdown of ATF3 using ATF3-siRNA suppresses the invasion ability of U373MG cells in vitro

**Figure 7:**
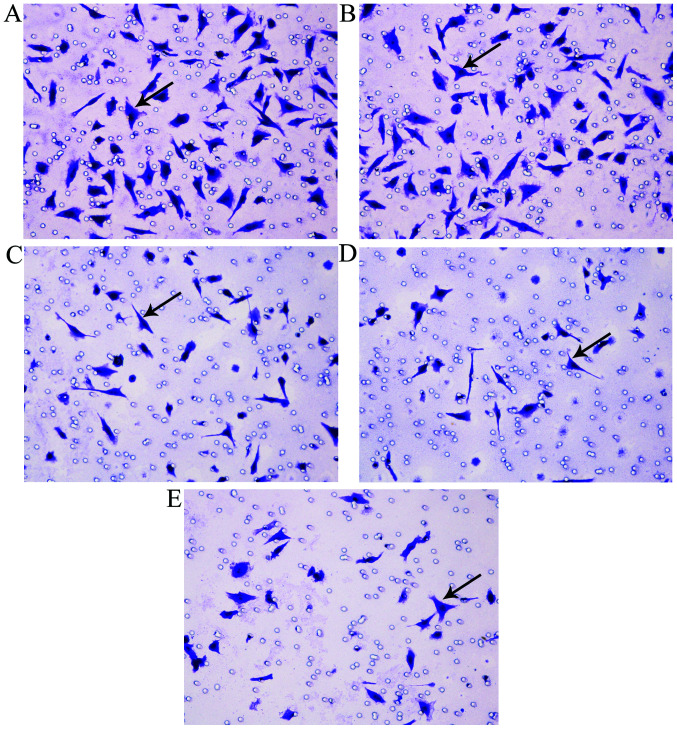
*In vitro* cell invasion assay. Arrowhead indicates U373MG cells that transversed the Transwell membrane (×400 magnification). (A) Untreated contorol group, (B) control-siRNA group, (C) activating transcription factor 3 (ATF3)-siRNA group, (D) cisplatin group, (E) ATF3 siRNA + cisplatin group. Cisplatin group and ATF3-siRNA + cisplatin group showed a marked decrease in the migratory cell number (P<0.05). The migratory cell number decreased more substantially in the cisplatin group and ATF3-siRNA + cisplatin treated group (P<0.05).

As the initial counts of cells added into the Transwell inserts were identical, changes in the invasion ability of the cells were evaluated by comparing the number of cells that penetrated the Transwell inserts to the initial number of cells present. The percentage of migrating cells (the number of cells that penetrated the Transwell inserts as a percentage of the initial number of cells) in each group was as follows: 76±6.5% in the untreated control group, 70±5.4% in the control-siRNA group, 43±3.8% in the ATF3-siRNA group, 26±4.2% in the cisplatin group and 25±3.2% in the ATF3 siRNA + cisplatin group. The difference between the untreated control and the control-siRNA group was not statistically significant (P>0.05). Compared to the untreated control group and the control-siRNA group, the percentage of migrating cells in the ATF3-siRNA group, the cisplatin group and the ATF3 siRNA + cisplatin group was markedly decreased (P<0.05). Compared with the ATF3-siRNA group, the percentage of migrating cells in the cisplatin group and the ATF3 siRNA + cisplatin group decreased even more substantially (P<0.05). There was no significant difference observed between the number of migrating cells in wells receiving ATF3 siRNA + cisplatin treatment and wells treated only with cisplatin (P>0.05; Fig. 7).

### Knockdown of ATF3 using ATF3-siRNA affects the mRNA levels of ATF3, maspin and MMP2 in U373MG cells in vitro

**Figure 8:**
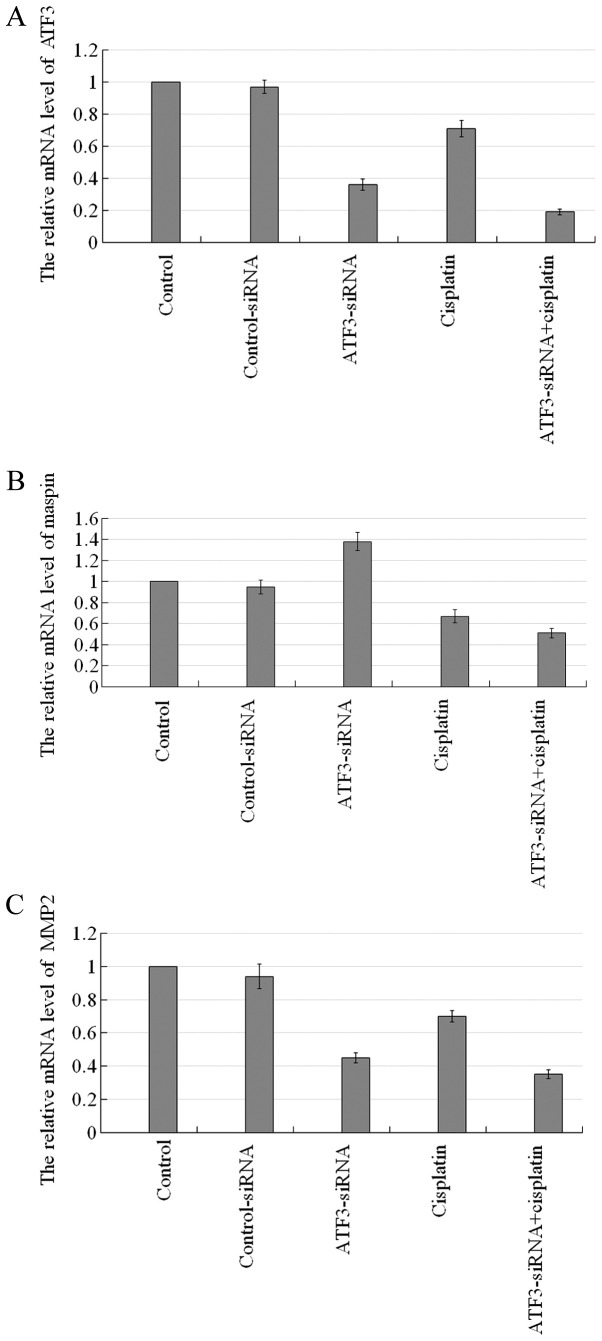
Effects of transfection with activating transcription factor 3 (ATF3) siRNA and of cisplatin on the relative mRNA levels of ATF3, maspin and matrix metalloproteinase 2 (MMP2). (A and C) The mRNA levels of ATF3 and MMP2 were consistent in each experimental group, with the relative mRNA expression of in the untreated control group and the control-siRNA group being the highest, followed by the the cisplatin group; the ATF3-siRNA group had much lower levels, and the ATF3-siRNA + cisplatin group the lowest. (B) The relative mRNa expression of maspin in the ATF3-siRNA group was highest, followed by the untreated control group and the control-siRNA group; the cisplatin group had much lower levels, and the ATF3-siRNA + cisplatin group the lowest.

A comparison between the untreated contorl group and the control-siRNA group revealed no significant difference (P>0.05), whereas a comparison between the other groups revealed significant differences (P<0.05; Fig. 8). The mRNA levels of ATF3 and MMP2 in each experimental group were basically at the same level, while they showed the highest expression level in the untreated control group and the control-siRNA group, followed by, in decreasing order, by the cisplatin group, the ATF3-siRNA group and the ATF3-siRNA + cisplatin group. The relative mRNA expression level of maspin was highest in the ATF3-siRNA group, followed by the untreated control group and the control-siRNA group, the cisplatin group, with the lowest expression being observed in the ATF3-siRNA + cisplatin group.

### Knockdown of ATF3 using ATF3-siRNA affects the protein levels of ATF3, maspin and MMP2 in U373MG cells in vitro

**Figure 9:**
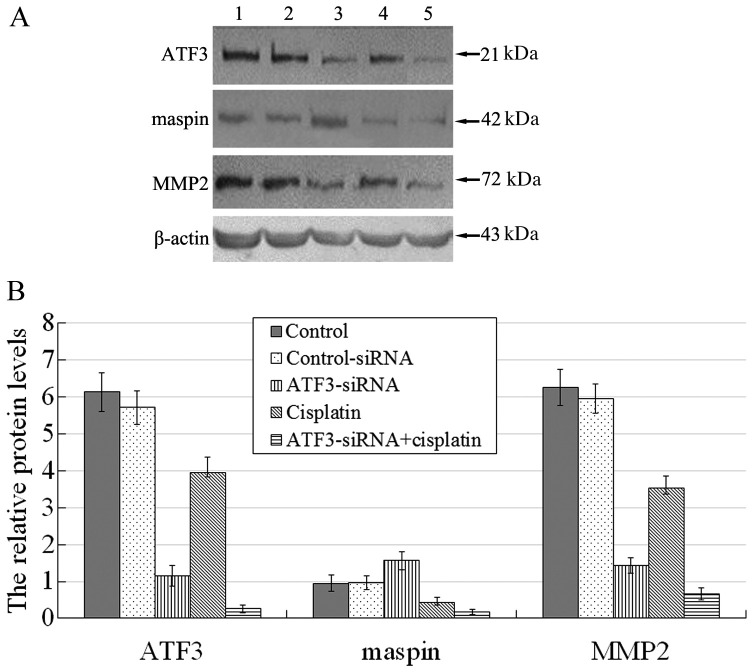
(A) Effects of transfection with activating transcription factor 3 (ATF3)-siRNA and of cisplatin on the protein levels of ATF3, maspin and matrix metalloproteinase 2 (MMP2). Lane 1, untreated control group; lane 2, control-siRNA; lane 3, ATF3-siRNA group; lane 4, cisplatin group; lane 5, ATF3-siRNA + cisplatin group. (B) Effects of transfection with ATF3 siRNA and of cisplatin on the relative protein levels of ATF3, maspin and MMP2. ATF3 and MMP2 protein expression was consistent in each experimental group and the expression of these 2 proteins (relative to β-actin) in the control group and control-siRNA group was the highest, followed by the cisplatin group; it was much lower in the ATF3-siRNA group, and lowest in the ATF3-siRNA + cisplatin group. By contrast, the relative protein expression of maspin was highest in the ATF3-siRNA group, followed by the untreated control group and control-siRNA group, much lower in the cisplatin group, and lowest in the ATF3-siRNA + cisplatin group.

A comparison between the untreated control group and the control-siRNA group revealed no significant difference (P>0.05), whereas a comparison between the other groups revealed significant differences (P<0.05; Fig. 9). The protein expression levels of ATF3 and MMP2 in each experimental group were basically at the same level, with the highest expression level being observed the untreated control group and the control-siRNA group, followed by, in decreasing order, by the cisplatin group, the ATF3-siRNA group and the ATF3-siRNA + cisplatin group. The relative protein expression level of maspin was highest in the ATF3-siRNA group, followed by the untreated control group and the control-siRNA group, the cisplatin group, with the lowest expression being observed in the ATF3-siRNA + cisplatin group.

### Knockdown of ATF3 using ATF3-siRNA inhibits the growth of U373MG xenograft tumors in vivo

**Figure 10:**
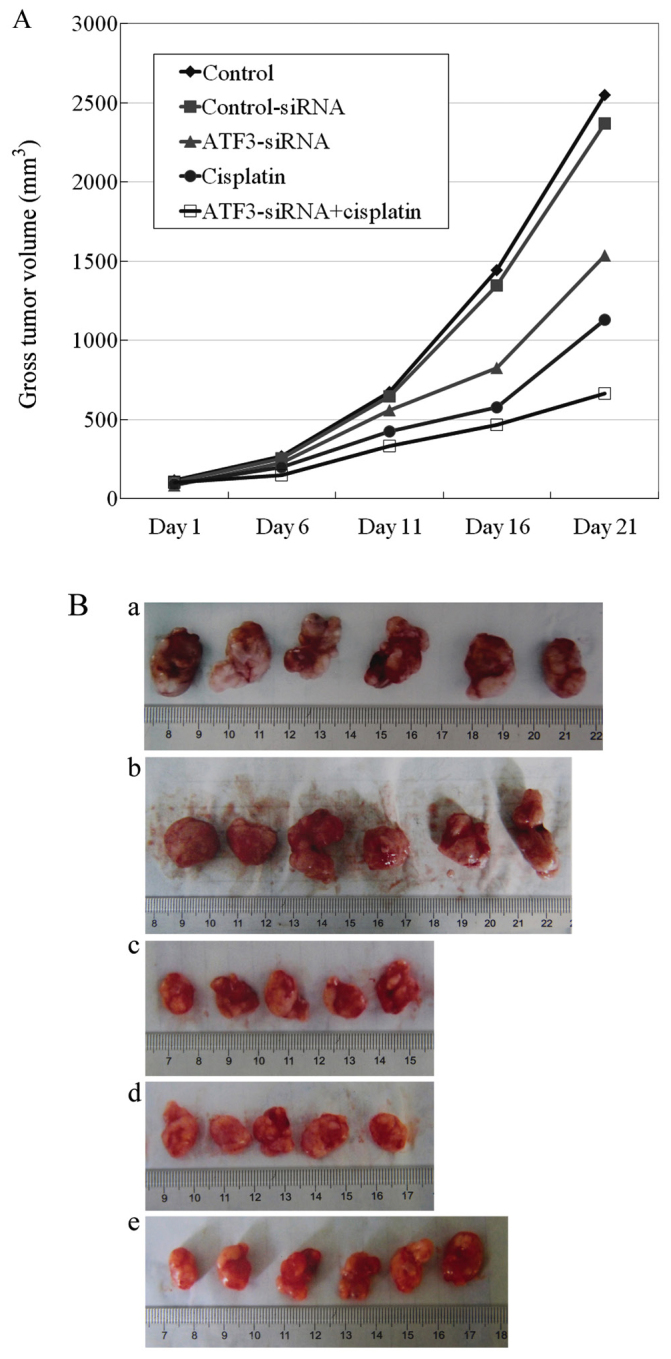
(A) Growth curves for U373MG tumor xenografts *in vivo*. From day 16 after treatment, compared to the vehicle control group and the control-siRNA group, the growth of U373MG cells *in vivo* was slower in the activating transcription factor 3 (ATF3)-siRNA group and tumor volume was significantly reduced (P<0.05). Growth in the cisplatin group was much slower, and tumor volume was much smaller (P<0.05) and tumor growth in the ATF3-siRNA + cisplatin group was the slowest, with the smallest volume (P<0.05). (B) Tumor size on day 21. (a) Vehicle control group, (b) control-siRNA group, (c) ATF3-siRNA group, (d) cisplatin group, (e) ATF3-siRNA + cisplatin group. Compared to the vehicle control group and the control-siRNA group, tumor volume was significantly reduced (P<0.05) in the ATF3-siRNA group. Tumor volume was much smaller (P<0.05) in the cisplatin group, and smallest in the ATF3-siRNA + cisplatin group (P<0.05).

Fifteen days after the subcutaneous injection of U373MG cells, the 40 nude mice were randomly assigned into 5 groups for treatment. Over the course of treatment several mice died: 2 in the vehicle control group, 2 in the control-siRNA group, 3 in the ATF3-siRNA group, 3 in the cisplatin group and 2 in the ATF3-siRNA + cisplatin group. The possible cause of death of the nude mice was that the individual difference of the nude mice caused them to have poor tolerance to the tumor load or they were allergic to the injected preparations. A significant difference was observed in tumor size between the ATF3-siRNA + cisplatin group and the other groups by day 6 post-treatment (P<0.05), while inter-group comparisons among other groups revealed no significant difference (P>0.05; Fig. 10). Intergroup comparisons among the vehicle control group, the control-siRNA group and the ATF3-siRNA group revealed no significant difference on day 11 post-treatment (P>0.05), while intergroup comparisons among other groups revealed significant differences (P<0.05). A comparison between the vehicle control group and the control-siRNA revealed no significant difference (P>0.05) on day 16, while intergroup comparisons among other groups revealed significant differences (P<0.05). The statistical interpretations for day 21 were the same as those for day 16. Tumor growth curves indicated that, compared with the vehicle control group and the control-siRNA group, tumor growth in the ATF3-siRNA group was attenuated, with a marked reduction in tumor size, although growth was slower and the tumor size smaller in the cisplatin group, and growth was slowest and tumor size smallest in the ATF3-siRNA + cisplatin group. The tumor growth inhibition ratio calculated by tumor weight (values in brackets) for day 21 with the vehicle control group as a negative control were: vehicle control group, 0% (1.58±0.35 g); control-siRNA group, 8.9±2.3% (1.44±0.31 g); ATF3-siRNA group, 41.1±3.7% (0.93±0.22 g); cisplatin group, 54.4±4.5% (0.72±0.17 g); and ATF3-siRNA + cisplatin group, 75.3±5.5% (0.39±0.11 g). A comparison between the vehicle control group and the control-siRNA group revealed no significant difference (P>0.05), whereas comparisons among other groups revealed significant differences (P<0.05; Fig. 10B).

## Discussion

ATF3 is a member of the ATF/CREB subfamily of the basic region-leucine zipper family. ATF3 regulates the expression of its target genes through complex mechanisms. It is an important regulatory factor for transcription, apoptosis, cell division and survival, and it plays an important role in controlling tumor invasion and migration (4–6). It has been reported that ATF3 is an oncogene and that it is overexpressed in human cancer tissues; for example, the gene copy number of ATF3 is significantly increased in breast cancers, which may be due to the increase in the ATF3 gene on chromosome 1q amplicon (the region with the largest increase in breast cancer) (12). In contrast to non-Hodgkin’s lymphoma and non-malignant tissue, a high level expression of ATF3 has been observed in Reed-Sternberg cells of patients with Hodgkin’s disease (2). Experimental results from the study by Pelzer *et al* (1) indicated that ATF3 was highly expressed in most prostate cancer cell lines, and that the overexpression of ATF3 induced the proliferation of prostate cancer cells and accelerated cell cycle progression from the G_1_ to the S phase. In accordance with these aforementioned experimental results, the present study demonstrated that glial cells in normal brain tissue also had a weak expression of ATF3, and that the expression of ATF3 was upregulated in glioma tissues, and increased with the increasing pathological grade of the glioma. ATF3 was also highly expressed in the glioblastoma cell line, U373MG, suggesting that a high expression level of ATF3 is closely related to the evolution of glioma and its malignant progression.

There is evidence in the literature that ATF3 may often promote the invasion and metastasis of cancer cell lines *in vitro* and *in vivo*. For example, Ishiguro *et al* (13) observed a high expression level of ATF3 in a highly metastatic subline of B16 melanoma cells and demonstrated that low-migratory B16 cells were changed into highly migratory cells by transfection with ATF3; however, ATF3 was not expressed in the parental B16 cell line. Bandyopadhyay *et al* (14) found that the transcription of ATF3 in a prostate cancer model was inhibited by the metastasis repressor, Drg-1; this suggests that ATF3 promotes metastasis in prostate cancer.

Related studies have also confirmed that ATF3 may be carcinogenic. For example, Ishiguro *et al* (15) abolished the expression of ATF3 using antisense oligonucleotides *in vitro*, and this reduced the adhesion and invasion of HT29 colon cancer cells. The effects of ATF3 antisense oligonucleotide intervention in mice inoculated subcutaneously with HT29 cells were investigated; the results revealed that the mice that received ATF3 antisense oligonucleotide intervention therapay, compared with the controls, were less likely to form tumors and had a longer average survival. A further demonstration of the effects of the selective knockdown of ATF3 expression by RNA interference was shown in the study by Janz *et al* (2) demonstrating that the proliferation of Hodgkin’s lymphoma cells was inhibited, with a reduced viability of the lymphoma cells, suggesting that ATF3 is related to the proliferation of cancer cells.

Our study supports the aforementioned studies. Our analysis of cell numbers following treatment of the U373MG cells *in vitro* (MTT assay) also suggested that following transfection with the plasmid expressing ATF3-siRNA, U373MG cell proliferation was reduced, cell cycle progression was inhibited and the growth inhibition rate of the U373MG cells gradually increased progressively with time. Our cell cycle analysis by flow cytometry revealed that, following transfection with ATF3-siRNA, the proportion of cells in the S phase was significantly reduced, and the percentage of cells arrested in the G_0_/G_1_ phase was significantly higher; the cell proliferation index was significantly lower than that for the control group. Our TUNEL staining results revealed that the proportion of U373MG cells showing signs of apoptosis increased significantly following transfection with ATF3-siRNA compared to the control group. Additionally, the invasion ability of the U373MG cells in Transwell culture was significantly weakened following transfection with ATF3-siRNA. Taken together, our experiments all directly confirm that transfection with ATF3-siRNA inhibits cell proliferation, reduce cell viability, arrests cell cycle progression, promotes apoptosis and impedes the migration of human glioblastoma cells, thereby indirectly supporting that ATF3 plays a facilitating role in the process of tumor invasion and metastasis.

Finally, our *in vivo* experiments confirmed that the repeated peritumoral injection of ATF3-siRNA compared with the vehicle-treated controls effectively inhibited the growth of U373MG tumor xenografts in nude mice. Treatment of xenograft tumors with ATF3-siRNA began to show effects at the 16th day of treatment; tumor growth curves revealed that tumor growth in the ATF3-siRNA group was attenuated and the tumor volume was significantly reduced.

The inhibitory effect on tumors was remarkable and similar effects were observed between ATF-siRNA and cisplatin treatments. The growth curves of the xenograft tumors of the nude mice indicated that tumor growth in the cisplatin group and the ATF3-siRNA + cisplatin groups was attenuated in a time-dependent manner; thus, the rate of increase in tumor volume decreased with time. Our growth data for the U373MG cells *in vitro* (MTT assay) indicated that cell growth was significantly suppressed in the cisplatin group and the ATF3-siRNA + cisplatin group, and the number of cells in both groups was significantly reduced compared with the first day. The reason for this difference in results may be that under *in vitro* conditions, human glioblastoma U373MG cells were more sensitive to cisplatin and ATF3-siRNA + cisplatin.

However, there is some evidence indicating the contrary, suggesting that ATF3 inhibits cancer formation. For example, the experimental results from the study by Lu *et al* (16) demonstrated that ATF3 suppressed Rasstimulated tumorigenesis *in vivo*. Another example is that the overexpression of ATF3 has been shown to reduce the size of tumor xenografts of HCT-116 human colorectal cancer cells placed subcutaneously in nude mice (17). The reason why these results differ materially may be due to the different experimental conditions and different experimental cell lines used. We confirm that, when faced with different cell types and driving factors, ATF3 plays different roles in the developmental process of cancer.

Maspin is a tumor suppressor gene discovered in 1994 (19). Maspin protein is moderately to highly expressed in a number of normal tissues, and plays an important role in inhibiting tumor growth, increasing cell adherence, reducing cell movement and invasion, and suppressing tumor angiogenesis; however, its expression is downregulated during tumor progression (18). Its loss is related to such factors as high malignancy, large tumor size, a high histological grade, lymph node metastasis, local recurrence, tumor development and a short survival time (19). The results of this study indicate that maspin protein was present both in the nucleus and cytoplasm and was highly expressed in the normal brain tissues. Its expression decreased in glioma from grade I to IV and negatively correlated with the pathological grade of the glioma. In U373MG cells, maspin protein was only moderately expressed and the expression rate was 47±6.4%. These results are consistent with the experimental results from the studies of both Zhang and Zhang (18) and Wang *et al* (20).

The important role of MMP2 in tumor neovascularization, cell infiltration and metastasis formation as a tumor-enhancing gene has been well-established (21–25,32,33). The results of our study demonstrated that the protein expression of MMP2 in glioma tissues was substantially higher than that in normal brain tissues, and the expression of MMP2 was also high in the U373MG cells. These results are consistent with the published study by Herbst *et al* (26).

In this study, we found that the ATF3 protein and mRNA levels in the human glioma tissues positively correlated with the expression of MMP2, and their expression was increased with the increasing pathological grade of the glioma. ATF3 expression negatively correlated with maspin expression, and the relative protein and mRNA expression of maspin was reduced with the increasing pathological grade of the glioma. *In vitro*, the expression of ATF3 and MMP2 in the U373MG cells was also consistent in each experimental group. Following transfection of the U373MG cells with ATF3-siRNA, the protein and mRNA levels of MMP2 decreased significantly, while the maspin protein and mRNA levels increased significantly; ATF3-siRNA downregulated the expression of MMP2, but upregulated that of maspin. These effects correspond with our experimental observations of human glioma tissues, that is, the expression of ATF3 showed the same trend as that of MMP2, but an opposite trend with maspin expression.

MMP2 is recognized as one of the strong cancer-promoting genes which promotes the invasion and metastasis of malignant glioma, and promotes tumor metastasis by degrading the extracellular matrix (21). The consistent association between ATF3 and MMP2 expression suggests that ATF3 exerts a similar function with MMP2 in some ways, and plays a role in promoting tumor metastasis. In contrast to MMP2, maspin is one of the classic tumor suppressor genes (27–29), and the opposite changes observed for ATF3 versus maspin expression suggest that the function of ATF3 opposes that of maspin, with ATF3 having a tumor-promoting role that is opposite to that of maspin.

The correlations between ATF3, maspin and MMP2 expression suggest that that they are placed at the intersection of molecular signaling pathways. One of the regulatory mechanisms of maspin involves p53 signaling; the study by Zou *et al* (30) demonstrated that infecting breast and prostate cancer cell lines with wild-type p53 adenovirus induced maspin expression. ATF3 and maspin both can bind p53 to induce a series of responses, so they are likely to act on the p53 pathway in glioblastomas. Maspin can also lead to a cell stress response through transcriptional regulation, and maspin re-expression may also lead to the suppression of several genes involved in the inflammatory response (27–30). ATF3 is not only an early stress response gene, but is also a hub of the cellular adaptive-response network for stress signals, and acts as the key regulation point that can be induced by a variety of factors (31). Thus, it may be speculated that ATF3 and maspin also act at the intersection of the stress response pathway and participate in the regulation of pro-inflammatory factors.

There are a number of sites on the MMP2 promoter which can be bound by regulatory elements, including binding sites for p53 and the cAMP response element binding protein (CREB) (32,33). ATF3 is a member of the CREB subfamily. In addition, MMP2 inhibits the inflammatory process, and MMP2 gene expression regulates multiple genes at the transcriptional and post-transcriptional level through the MAPK pathway (32,33); the MAPK and p53 pathways are closely related to ATF3 (1,34). Thus, it can be speculated that the correlation in the expression of MMP2 and ATF3 acts in a complex linkage to regulate signaling pathways and the expression of inflammatory cytokines.

Contrary to our results, there are studies describing that ATF3 inhibits the expression of MMP2. Stearns *et al* (35) found that ATF3 suppressed MMP2 expression by directly binding with the MMP2 promoter, and Yan *et al* (36) demonstrated that ATF3 exerted its inhibitory effect by interfering with the transcriptional activation of MMP2 through p53. These inconsistent experimental results may be related to different conditions and apparatus used in different laboratories, and perhaps with MMP2 and ATF3 in different tissues through different signal transduction pathways.

In this study, the inhibitory effects of ATF3-siRNA were observed in the human glioblastoma cell line, U373MG, *in vitro* and in tumor xenografts *in vivo*. This indirectly suggests that ATF3 exerts promoting effects on the development and invasion process of glioblastoma, although the most relevant target genes and the corresponding signaling pathways through which ATF3 promotes glioblastoma cells to invade, and factors regulating ATF3 in glioblastoma during the invasion process have not yet been fully elucidated. Further studies are required to clarify this, in order to identify new molecular targets and develop new treatment strategies for the treatment of glioblastoma.

## Figures and Tables

**Table I:** Protein expression of ATF3, maspin and MMP2 in normal brain tissues and glioma tissues of each grade.

	n	ATF3 n (%)	Maspin n (%)	MMP2 n (%)
Normal brain tissues	13	2 (15.4)	13 (100)	1 (7.7)
Gliomas tissues	100	72 (72)^a^	53 (53)^a^	76 (76)^a^
WHO grading				
I	15	4 (26.7)	12 (80)^a^	4 (26.7)
II	32	18 (56.3)^a^	22 (78.1)^a^	20 (62.5)^a^
III	30	28 (93.3)^a^	13 (43.3)^a^	29 (90.6)^a^
IV	23	22 (95.7)^a^	6 (26.1)^a^	23 (100)^a^

Positive expression status of ATF3, maspin and MMP2 in normal brain tissues and glioma tissues was assayed by immunohistochemistry (numbers in parentheses indicate positive rates, numbers outside parentheses indicate positive case numbers). The positive protein expression levels of ATF3 and MMP2 in the glioma tissues were significantly higher than those in normal brain tissues (72 and 76%, respectively, P<0.05), but the positive protein expression rate of maspin in the glioma tissue was lower than that in the normal brain tissue (53%, P<0.05). In grade II–IV gliomas, the expression of ATF3 and MMP2 was higher than that in normal brain tissue (P<0.05). In all grades of glioma tissue, maspin protein expression was lower than that in normal brain tissue (P<0.05).

a Significant differences compared with normal brain tissue (P<0.05). ATF3, activating transcription factor 3; MMP2, matrix metalloproteinase 2.
